# On the Use of Hearing Aid Algorithms in Industrial Noise: Balancing Speech Intelligibility and Hearing Protection

**DOI:** 10.1177/23312165261478230

**Published:** 2026-09-06

**Authors:** Solenn Ollivier, Fabien Bonnet, Alexis Pinsonnault-Skvarenina, Christian Giguère, Jérémie Voix, Hugues Nélisse

**Affiliations:** 1 14849 École de technologie supérieure, Université du Québec, Montréal, QC, Canada; 2 97890 Institut de recherche Robert-Sauvé en santé et sécurité du travail, Montréal, QC, Canada; 3 School of Rehabilitation Sciences, 12369 Université Laval, Québec, QC, Canada; 4 Centre for Interdisciplinary Research in Rehabilitation and Social integration (CIRRIS), 113463 Centre Intégré Universitaire de Santé et de Services Sociaux de la Capitale-Nationale (CIUSSS-CN), Québec, QC, Canada; 5 151172 School of Rehabilitation Sciences, University of Ottawa, Ottawa, ON, Canada

**Keywords:** hearing protection devices, hearing aids, communication in noise, speech intelligibility, signal processing algorithms, noise exposure, industrial noise, hearing loss

## Abstract

Hearing protection devices (HPDs) are widely used to mitigate the risks of noise-induced hearing loss in industrial environments. However, conventional HPDs can often hinder communication for workers with hearing loss. While those workers may rely on hearing aids in their daily lives, the potential benefits and associated risks of using such devices in noisy workplaces remain unexplored. This study evaluated the effects of different hearing aid signal processing algorithms on speech intelligibility and estimated *noise exposure* under industrial noise conditions to characterize the balance between communication and protection needed to develop solutions for workers with hearing loss. A digital hearing protection research platform integrating in-ear receivers and external microphones was configured as a real-time soundcard. Typical hearing aid algorithms, including linear amplification, Wide Dynamic Range Compression (WDRC), digital noise reduction (DNR), and compression limiting, were implemented in different configurations. Two groups, one with normal hearing and one with hearing loss, completed the Hearing in Noise Test (HINT) with a wood-working factory noise at two presentation levels, along with subjective ratings of *listening effort* and *voice clarity and naturalness*. *Noise exposure* for each configuration was also estimated. Results showed significant effects of signal processing strategies and noise presentation levels on both intelligibility and noise exposure. Combining DNR, WDRC and compression limiting showed promising results to balance intelligibility and noise exposure, particularly for participants with hearing loss. The findings emphasize the trade-off between auditory benefit and exposure risk, and call for greater awareness regarding the use of hearing devices in industrial noise.

## 1. Introduction

Noise exposure is one of the most common occupational hazards. Prolonged or repeated exposure to loud sounds can cause permanent damage to the auditory system, leading to noise-induced hearing loss (NIHL). Worldwide, around 16% of hearing loss in adults is caused by noise exposure (Nelson et al., 2005). In Canada, among the 43% of Canadian workers who have been exposed to noise, 38% had some degree of hearing loss (Feder et al., 2015). In the Canadian province of Quebec, a recent retrospective cohort study found that the incidence of NIHL remained between 4 and 7% from 1980 to 2019, with a slight increase since 2000, and that most exposed workers faced levels above the 85 dBA/8h regulatory limit (Dahan et al., 2025). NIHL compromises the ability to hear, distinguish, and localize sounds, increasing communication difficulties, especially in noisy environments (Sheppard et al., 2020). In the workplace, NIHL is associated with increased stress and fatigue (Morata et al., 2005), reduced concentration and a higher risk of injury (Cantley et al., 2015; Leroux et al., 2018; Picard et al., 2008). In Quebec, 12% of work-related injuries have been attributed, at least partially, to NIHL and noise exposure (Picard et al., 2008).

The preferred approach to reduce workers’ noise exposure is to identify the source of noise emission and to remove or reduce it, through engineering controls (Canadian Centre for Occupational Health and Safety, 2023, 2024b; Meinke et al., 2022). When such controls are not technically or economically feasible, administrative measures, such as task rotation or scheduling noisy operations during off-peak hours, may be implemented. If residual noise exposure remains above acceptable limits, workers must wear hearing protection devices (HPDs), which attenuate ambient sounds when adequately fitted and used. For workers with hearing loss, passive HPDs often exacerbate communication difficulties and affect the perception of warning signals, compromising safety and working efficiency (Arz et al., 2019; Giguère & Berger, 2016; Giguère et al., 2010). Level-dependent HPDs have shown promising benefits in speech intelligibility in noise for those workers; while they do not always enhance speech intelligibility, when compared to passive HPDs, they do not worsen it and can prevent overprotection (Giguère et al., 2014; Nakashima & McDavid, 2018). However, they usually provide generic signal processing, so the amplification provided is not tailored to the worker’s hearing loss in each ear (Leroux et al., 2018), highlighting the potential value of customized solutions for HPDs by integrating hearing aid functionalities.

To improve listening and communication ability in their daily life, people with hearing loss typically wear hearing aids. In some contexts, these devices may also be considered for use in noisy occupational environments to improve speech intelligibility and detect important machinery sounds (Leroux et al., 2018), even though they are primarily designed to be used in daily listening environments and are not intended for high-level industrial noise. Modern hearing aids typically implement several signal processing strategies, such as wide dynamic range compression (WDRC), digital noise reduction (DNR), and compression limiting, also known as automatic gain control output (AGCo). These algorithms have different objectives and may influence speech intelligibility, listening effort, and noise exposure in different ways. For example, although DNR is not typically considered as a hearing protection feature, it may reduce overall noise exposure for workers who require amplified speech to maintain audibility in noisy environments. More specifically, traditional DNR has the capability of attenuating background noise while maintaining speech at audible levels. Even though DNR generally does not improve speech intelligibility, it has been reported to improve perceived listening effort (Brons et al., 2013, 2014; Desjardins & Doherty, 2014; Sarampalis et al., 2009). Therefore, this could result in a significant reduction in overall noise exposure. Additionally, while the effects of compression limiting and WDRC on speech perception and sound localization have been studied in typical daily environments (Souza, 2002), few studies have examined their performance in industrial workplace noise (Ollivier et al., 2025). Typical hearing aid parameters, such as the compression limiting thresholds, must be modified when integrated into advanced protection devices to safely manage daily sound exposure in noisy occupational settings. In return, such adaptations may affect speech perception performance (Ollivier et al., 2025; Souza, 2002) and/or listening comfort, and more research is needed to better understand the impact of these potential interactions. At the same time, there is concern that amplification provided by hearing aid signal processing in already loud environments could increase the risk of NIHL. One study reported that hearing aids based on linear amplification could amplify already loud sounds to levels well above recommended exposure limits (Dolan & Maurer, 1996). Moreover, Macrae (1994) demonstrated that the risk of hearing loss aggravation attributed to linear hearing aid use depends on the user’s hearing thresholds. Despite major advances in hearing aid design and the widespread use of non-linear algorithms, such findings highlight the long-standing concern that hearing aids may expose users to unsafe noise levels, increasing the risk of additional hearing damage (Leroux et al., 2018).

These uncertainties are reflected in the diversity of occupational health recommendations regarding the use of hearing aids and hearing protection in noisy environments for workers with hearing loss. To protect workers from further hearing loss, the Canadian Centre for Occupational Health and Safety (CCOHS) recommends using HPDs, with or without hearing aids, whenever noise levels approach occupational exposure limits (Canadian Centre for Occupational Health and Safety, 2024a). In the United States, the Occupational Safety and Health Administration (OSHA) stated that hearing aids should not be considered as HPDs (Fairfax, 2004). Instead, noise-exposed workers with hearing loss are advised to wear their hearing aids turned on, combined with appropriate hearing protection, to ensure both awareness and protection. Other solutions typically observed in noisy workplaces include using hearing aids turned off, turned on but with a reduced volume, or with noise reduction settings (Leroux et al., 2018). The CSA Group suggests the use of level-dependent HPDs as a possible measure, highlighting that hearing aids combined with HPDs should only be considered on a case-by-case basis following an audiologist’s guidance (Canadian Standards Association Group, 2022).

This diversity of recommendations reflects the absence of consensus among hearing health professionals regarding the safest and most effective practice, largely due to the lack of empirical research on hearing aid use in industrial settings. In particular, it is necessary to better understand how typical hearing aid signal processing strategies can be adapted to occupational constraints and how these modifications will affect speech intelligibility and cumulative noise exposure in noisy occupational environments.

A novel technological approach is used in this research. An electronic research platform to be used as a “protective hearing aid” (“prothèse protégeante” in French, dubbed “ProPro”) was developed and used to investigate the use of hearing aid signal processing in occupational noise conditions. The ProPro platform integrates multiple functionalities within a single system, including digital audio signal processing functionalities and custom amplification based on individual hearing profiles, as well as a passive hearing protection functionality. In contrast to conventional hearing aids, the platform employs an output limiting strategy that is adapted to occupational exposure constraints, enabling the evaluation of established hearing aid signal processing strategies within a hearing protection context. Additionally, the research platform allows accurate estimation of in-ear noise exposure through calibrated signal recordings. Using this platform, the present study examined how common hearing aid signal processing strategies, adapted for occupational constraints, affect speech intelligibility and overall noise exposure in industrial noise. The objectives of this study were twofold: (1) to assess how different algorithms adapted to occupational constraints affect speech intelligibility for listeners with and without hearing loss in industrial noise, and (2) to examine how these algorithms influence overall noise exposure levels. By jointly considering speech intelligibility and noise exposure, this work provides a first step toward evidence-based recommendations for the design of hybrid protection devices combining hearing aid and hearing protection functionalities for use in occupational noise environments.

## 2. Materials and Methods

### 2.1. Development of the ProPro Research Platform

#### 2.1.1. Hardware

**Figure 1.**
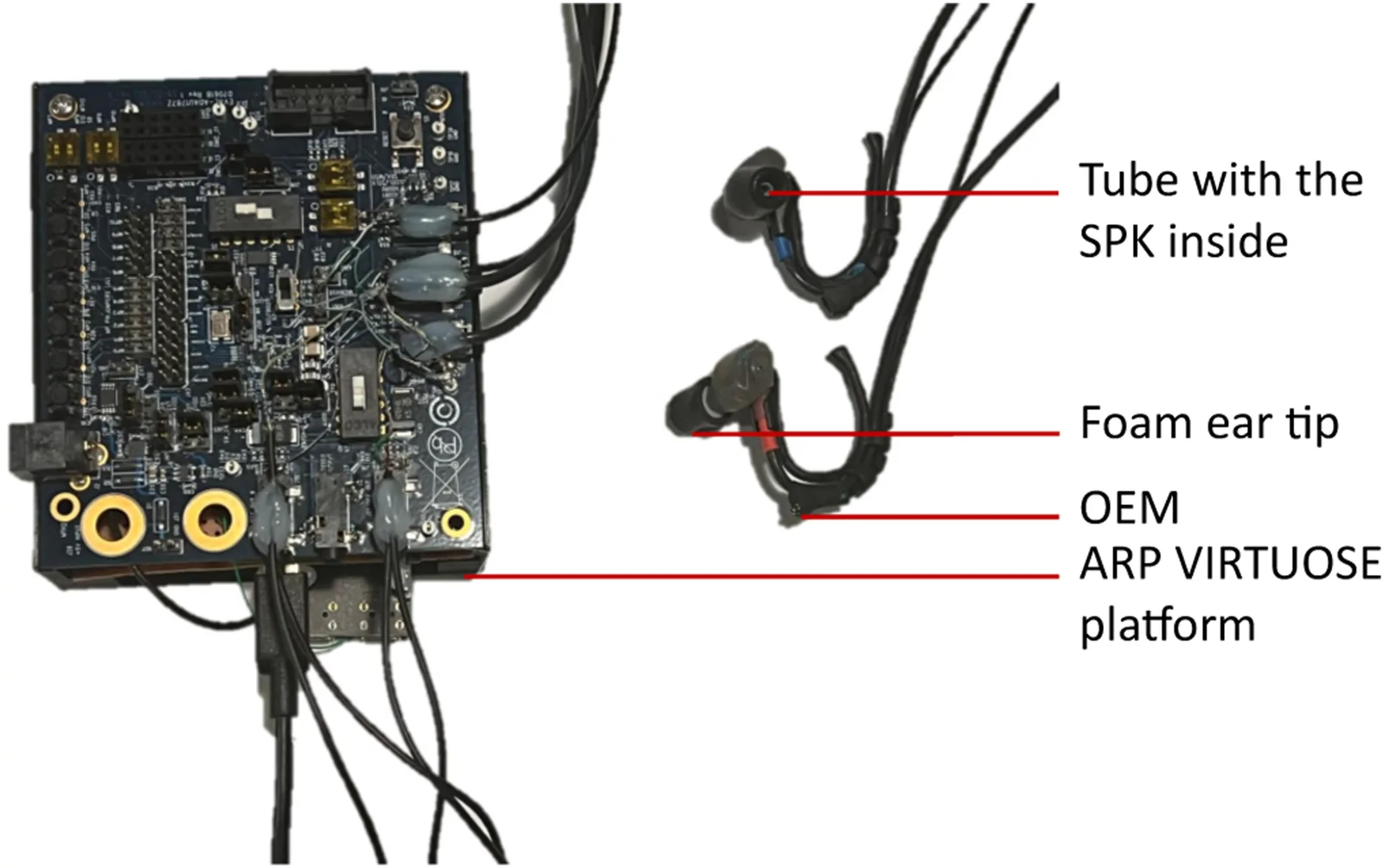
The ARP VIRTUOSE as designed for the ProPro research platform. Each earpiece was mounted with a foam eartip.

**Figure 2.**
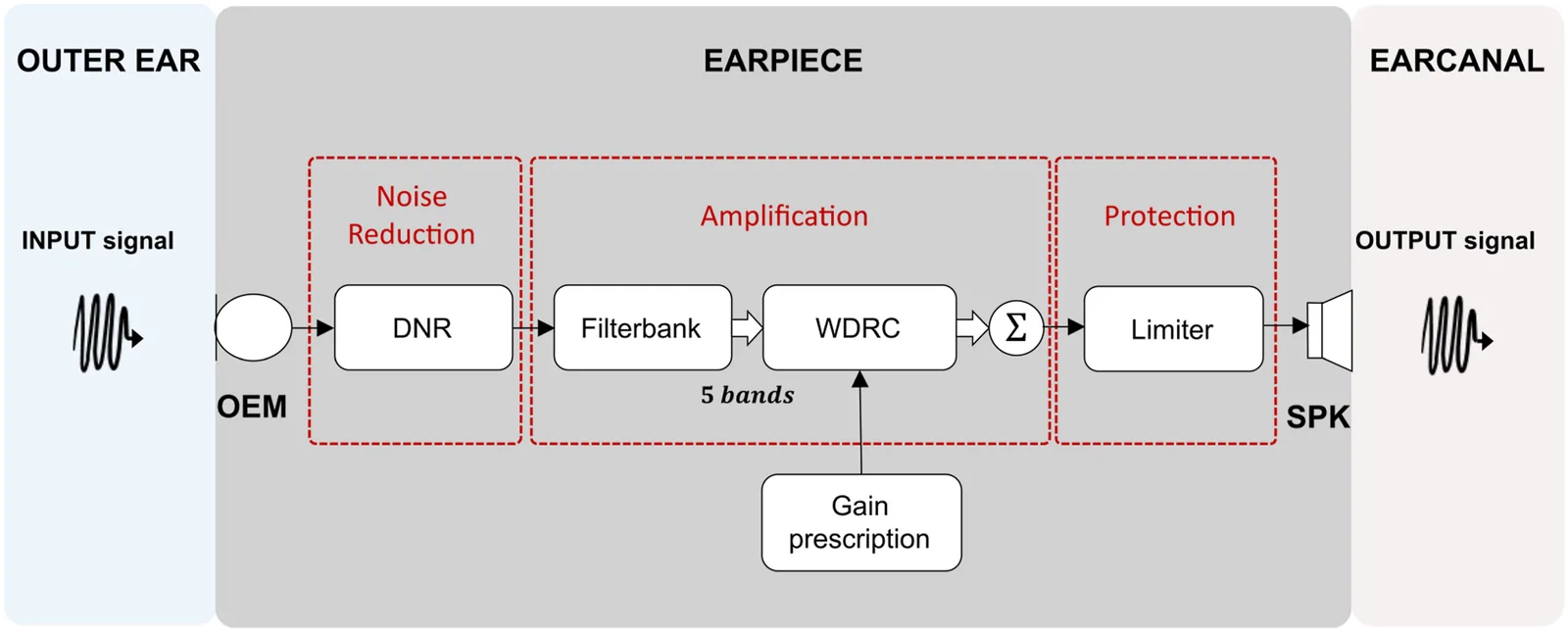
Block diagram showing a configuration of the implemented algorithms. WDRC parameters were adjusted according to the prescribed gain.

The research platform used to develop ProPro was the Auditory Research Platform (ARP), “VIRTUOSE” version, developed within the EERS-ÉTS Industrial Research Chair in In-Ear Technologies (CRITIAS) and illustrated in Figure 1. It integrates a Teensy 4.1 micro-controller (Sherwood, OR, USA) and an ADAU1787 codec from Analog Devices (Wilmington, MA, USA). Its flexible configuration allows it to function both as a sound card and as a standalone processing device. A pair of earpieces, equipped with 3M Comply^TM^ short ear tips (St. Paul, MI, USA), was connected to the ARP, as illustrated in Figure 1, providing electroacoustic hearing protection through compression limiting, and hearing aid functionalities through multichannel WDRC and real-time signal processing. As shown in Figures 1 and 2, each earpiece included an outer-ear microphone (OEM) to pick up external sounds and an internal miniature receiver (SPK) to play back the processed signal. The references for the components were FG-23652-D65 for the analog microphones and RAF-32902 for the receivers, all manufactured by Knowles (Itasca, IL, USA). It can be noted that passive attenuation can be achieved when earplugs are fitted with foam ear tips; however, passive hearing protection was not quantified nor analyzed in the present study.

The ARP was operated as a multichannel audio soundcard connected to a personal computer. The use of a specific platform for the speech intelligibility test, described in section *Speech intelligibility assessment*, imposed a limitation on the setup: only the receivers were used to play the pre-recorded and processed signals, simulating experimental conditions where sound transmission through the passive attenuation of the earpiece is negligible.

#### 2.1.2. Hearing Loss Prescription and Signal Processing

Signal processing for the active hearing protection and hearing aid functionalities of the ProPro platform was implemented in Matlab (Natick, MA, USA) using a typical hearing aid signal processing architecture. Figure 2 illustrates one configuration of the processing chain, which includes DNR, either WDRC or linear amplification for gain adjustment, and a final stage of compression limiting for protection against intense sounds.

For noise reduction, a Wiener-filter-based DNR algorithm was implemented (Loizou, 2013). The parameters were empirically adjusted to improve the signal-to-noise ratio (SNR) while minimizing musical noise. This traditional DNR algorithm, commonly used in hearing aids, has been shown to perform better than other DNR algorithms for speech intelligibility in noise, and, under ideal but artificial conditions can nearly restore intelligibility (Kates, 2017; Madhu et al., 2013). Preference for DNR combined with slow-acting compression has also been reported (Rallapalli et al., 2022). While more advanced approaches based on Deep Neural Network (DNN) may offer measurable benefits, they were not considered in the present study.

To enable frequency-specific amplification, the input signal was divided into five channels using an all-pass crossover filter bank (D’Appolito, 1987). The crossover frequencies (450, 900, 1800, and 3575 Hz) were selected so that each channel carried approximately 20% of speech cues according to the Speech Intelligibility Index (SII) (American National Standards Institute, 1997). The number of channels, set to five, provided a compromise between gain adjustment flexibility (Kates, 2010; Yund & Buckles, 1995) and acceptable processing delay (Galster & Galster, 2011). The amplification was then applied independently to each channel based on the participant’s hearing profile.

Insertion gains were prescribed using the NAL-RP procedure (Byrne, 1991; Dillon, 2012) and applied for a 65 dB input level, referred to as the Level of Gain Application (LGA). Equivalent levels for the LGA in each channel were estimated by filtering a calibrated broadband speech-shaped noise, set to the LGA, through the filter bank and by computing channel-specific Root Mean Square (RMS) levels. The amplification gain for each channel was calculated by linearly interpolating the prescribed gain between frequency channels.

**Figure 3.**
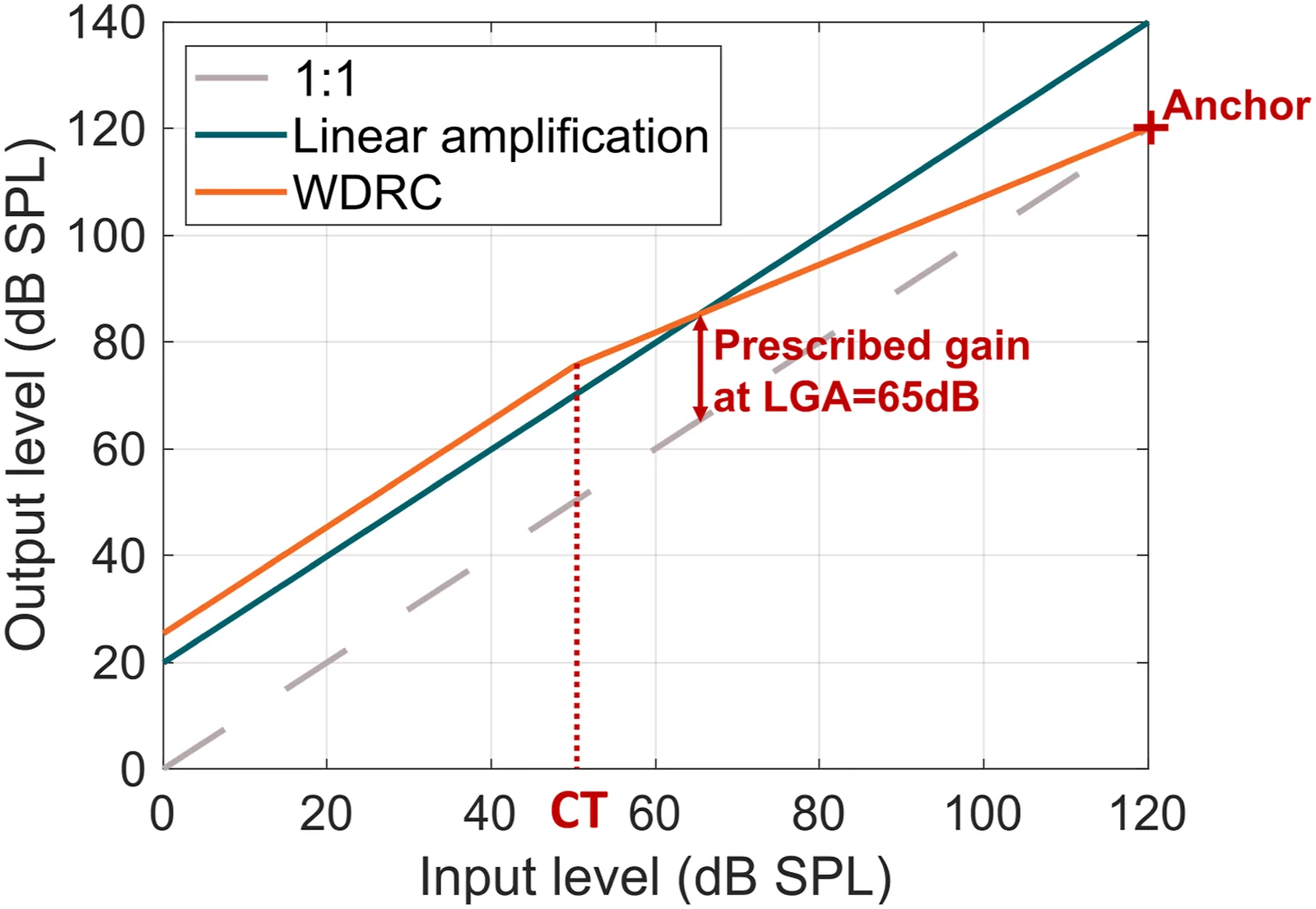
(Color online) Input–output curves for linear amplification and WDRC. WDRC provides more gain than linear amplification at low input levels and less gain at higher input levels. *Note.* For multichannel WDRC, equivalent levels for CT, LGA and Anchor were estimated in each channel to meet the broadband target (see text for details).

Two amplification strategies were tested: linear gain and WDRC. For linear amplification, the computed gain for each channel was applied equally across all input signal levels, which could over-amplify high-level inputs. Linear amplification served as a control to isolate the effect of WDRC on speech intelligibility and estimated noise exposure. WDRC adjusted output levels depending on the input levels. Sounds with levels below a 50 dB compression threshold (CT) were amplified with a given gain to improve audibility. Sounds with levels above the CT received less gain according to the compression ratio (CR). The CT value was chosen following NAL-NL1 recommendations (Dillon, 1999; Keidser et al., 2011). The applied gain was smoothed over time using attack (AT) and release (RT) times to avoid abrupt fluctuations (AT = 10 ms, RT = 500 ms). The RT was chosen to improve speech intelligibility in noise (Kates, 2010; Shetty & Raju, 2019). An anchor point was defined for each channel at an input level of 115 dB SPL overall, with the corresponding output level set equal (no gain applied). The anchor point was used, alongside with the prescribed gain at the LGA and the CT, to compute the gain below the CT, and the compression ratio in each channel. The anchor point level was set low enough to avoid amplification above the threshold of discomfort, while remaining sufficiently high to prevent negative input-output slopes above the CT, particularly for greater degrees of hearing loss. Both the equivalent level for each channel of the CT and anchor point were computed using the same procedure as was used for the LGA. For each channel, the gain adjustment can be illustrated with an input-output curve, as depicted in Figure 3.

Finally, because the platform was designed for protective use, a compression limiting algorithm was used to ensure protection against very intense sounds. Compression limiting was systematically applied in all tested configurations, including those without amplification or noise reduction, immediately before playback through the receiver as shown in Figure 2. It used a 90 dBA diffuse-field equivalent threshold, adapted to occupational constraints, and lower than limiting thresholds of conventional hearing aids which are typically based on the listener’s uncomfortable loudness level. This methodological difference may influence speech intelligibility outcomes relative to those reported in the hearing-aid literature. The compression limiting compression ratio was 10:1, and short time constants were used (AT=5 ms, RT=50 ms) to attenuate a sudden increase in sound level. With such parameters, the time-weighted exposure was expected to remain below safe levels, while maintaining good speech intelligibility even if short-term noise peaks exceeded the threshold. An A-weighted output limiting level was chosen to make the noise exposure from the device directly comparable to the occupational noise exposure limits.

### 2.2. Participants

For this study, approved by the ethics committee of the École de technologie supérieure (#H20230502), 18 participants aged between 18 and 80 years were recruited. Participants’ characteristics are presented in the Results section and summarized in Table 1. After giving informed consent, participants completed a questionnaire regarding general health and otologic history. They were required to have no history of ear surgery and no unmedicated attention deficit hyperactivity disorder. Additional inclusion criteria were (1) normal otoscopy for both ears; (2) no air-bone gap exceeding 15 dB HL at each tested frequency; and (3) native French language proficiency.

**Table 1.** Summary of Participants’ Sex, Age and *PTA*_4_ (dB HL) for the Normal Hearing (NH) and Hearing Loss (HL) Groups

​	HL (n=8)	NH (n=10)
**Sex**, n (%)		
Male	7 (87.5%)	3 (30%)
Female	1 (12.5%)	7 (70%)
**Age (y.o.)**, Mean (SD)	60 (16)	46 (13)
**PTA**_**4**_ **(dB HL)**, Mean (SD)		
Better ear	33 (13)	8 (6)
Worse ear	42 (15)	10 (6)

Each participant underwent an audiological assessment. Bilateral otoscopy ensured the absence of external ear canal or tympanic membrane abnormalities. Air- and bone-conduction pure-tone thresholds were measured bilaterally using an Interacoustics AC-40 audiometer (Middelfart, Denmark) in an Eckel double-walled, soundproofed, and electrically shielded booth (Morrisburg, ON, Canada), adhering to ANSI S3.1 ambient noise standards (American National Standard Institute, 1999). Testing was conducted with Telephonics TDH39P headphones (Farmingdale, NY, USA) and RadioEar B71 bone transducer headset (Middelfart, Denmark). The audiometer and earphones underwent electroacoustic calibration in accordance with ANSI S3.7 (American National Standards Institute, 1995). Hearing thresholds were determined using the Hughson-Westlake procedure at frequencies of 250, 500, 1 k, 2 k, 3 k, 4 k and 6 kHz for air conduction, and at 500, 1 k, 2 k and 4 kHz for bone conduction (Carhart & Jerger, 1959). Participants were assigned to two groups based on their better ear’s pure-tone average at 500, 1 k, 2 k, and 4 kHz (PTA_4_): the normal hearing (NH) group (PTA_4_ ≤ 20 dB HL) and the hearing loss (HL) group (PTA_4_ > 20 dB HL). While this study primarily focused on exploring potential solutions for participants with hearing loss, participants with no hearing loss were also included. The developed prototype can be characterized as a level-dependent HPD, a category of HPDs also used by workers without hearing loss.

### 2.3. Speech Intelligibility Assessment

Speech intelligibility was assessed using the French-Canadian adaptation of the Hearing in Noise Test (HINT) (Vaillancourt et al., 2005). The HINT measures speech reception thresholds (SRTs) in both quiet and noise. The SRT in noise is the SNR at which the participant can recognize 50% of the presented speech material (Nilsson et al., 1994). The speech material consisted of 12 lists of 20 phonetically balanced sentences. The test was administered through the Matlab Speech Test Environment (MSTE), developed by Ellaham et al. (2014), which uses Head Related Transfer Functions (HRTFs) to simulate spatial presentation under earphones. Participants’ hearing thresholds were logged in the MSTE interface, and signal processing algorithms described in section *Hearing loss prescription and signal processing* were implemented. Finally, processed speech and noise signals were played through the receivers of the platform’s earphones, the ARP being used as a stereo sound card. The HINT was also administered in the double-walled, soundproofed, and electrically shielded booth.

**Figure 4.**
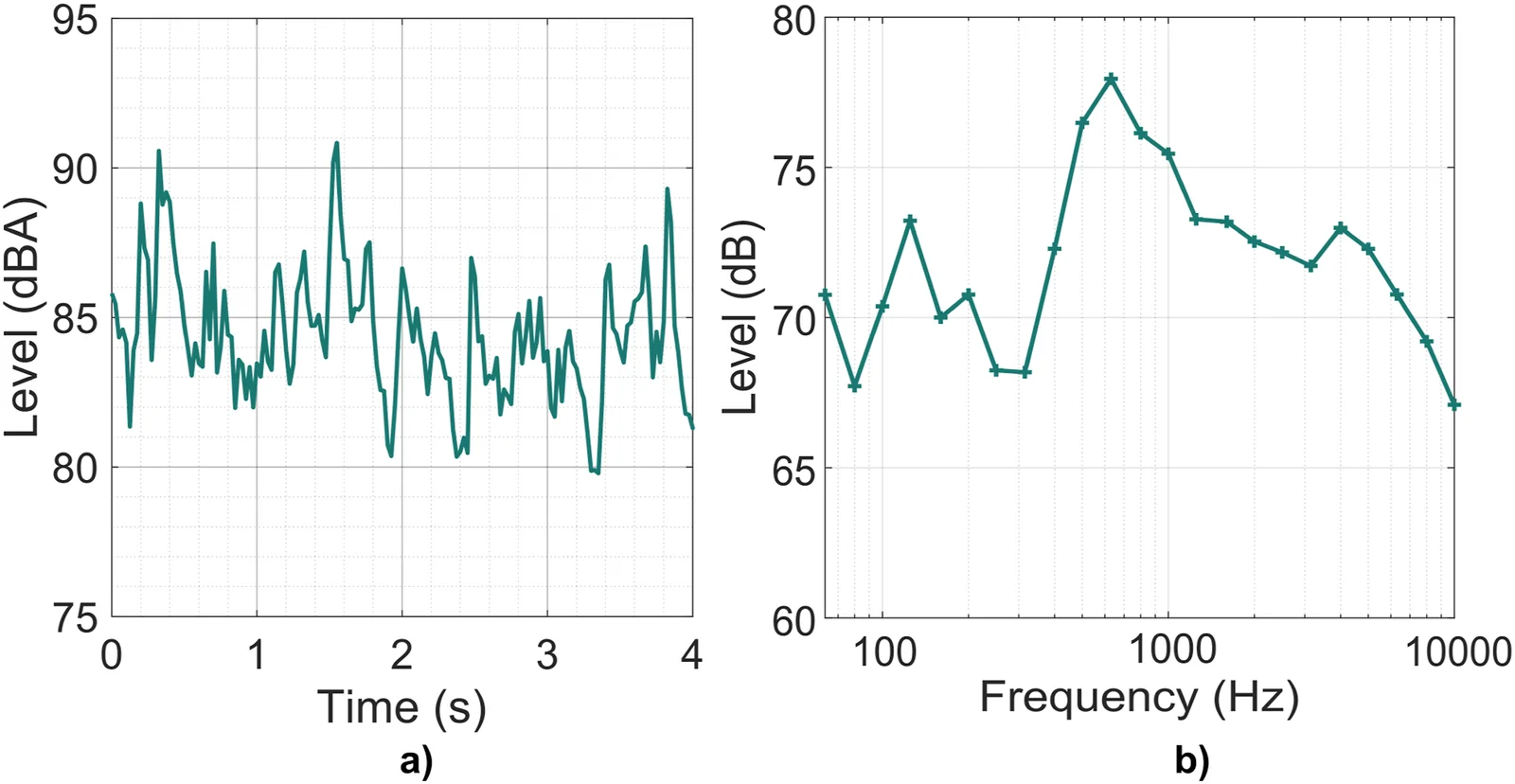
(A) Temporal and (B) spectral characteristics of an extract of the wood-working factory noise. Illustrative temporal characteristics represent the time evolution of the equivalent A-weighted level as measured every 25 ms for a 85 dBA signal.

Participants completed the HINT through MSTE under 8 scenarios (4 signal processing strategies x 2 noise presentation levels). Four hearing aid algorithms (linear amplification, WDRC, compression limiting, and DNR) were tested in various combinations. Prior to implementing these algorithms, the device was first calibrated for transparent signal processing (i.e., 0-dB insertion gain) on a head and torso simulator (HATS) GRAS 45BB7 (Holte, Denmark) for simulated diffuse-field sound presentation through the receivers of the ARP. The tested combinations, which all included compression limiting, were (a) transparent signal processing (referred to as “NoAmp”), (b) multichannel linear amplification (LinAmp), (c) multichannel WDRC (Wdrc) and (d) DNR before amplification through multichannel WDRC (DnrWdrc). The HINT sentences were mixed with random samples of a wood-working factory noise, whose characteristics are illustrated in Figure 4. The noise was presented at diffuse-field–equivalent levels of 65 dBA and 85 dBA and started two seconds before the onset of each sentence to allow the hearing aid algorithms to adapt. The levels of 65 dBA and 85 dBA were chosen to assess the performance of hearing aid algorithms in both loud and quieter working environments. At such levels, the passive sound pathway contribution to noise exposure would be negligible, given the attenuation provided by 3M Comply^TM^ short ear tips with an average noise reduction rating above 29 dB. In-ear sounds would be dominated by the electronic sound pathway, given the stimulus level and processing functions, supporting the sole use of in-ear receivers for stimuli presentation. For each scenario and participant, a random sentence list was selected and the sentences within each list were presented in random order. Participants were instructed to repeat each sentence, or any words they could understand. Before testing, they completed a practice session in the scenario at 65 dBA without any amplification (NoAmp).

SRTs were measured following the HINT adaptive procedure implemented in MSTE, adjusting the SNR based on the participant’s previous response by increasing or reducing the sentence level with a fixed-level noise. The speech level was varied in 4-dB steps for the first five sentences and in 2-dB steps thereafter. The SRTs were calculated as the mean SNR of sentences 5 to 20, with a theoretical 21^*st*^ sentence for which the SNR was estimated from the participant’s response to sentence 20 (Ellaham et al., 2014).

### 2.4. Subjective Ratings of Speech Quality and Listening Effort

After obtaining SRTs, subjective perception of the processed signals was evaluated. Two questions were adapted from the Speech, Spatial and Qualities of Hearing Scale (SSQ), version 5.6 (Gatehouse & Noble, 2004), which was originally designed to assess hearing disability across domains such as sound quality and speech intelligibility. The selected items targeted: (A) the perceived listening effort and (B) the speech clarity and naturalness. The two statements in French translated to “A. Understanding this sentence doesn’t require much effort” and “B. The voice sounds clear and natural”. For this evaluation, a single prerecorded French sentence (“J’aime les couchers de soleil”) was processed with each signal processing algorithm and presented with a 0 dB SNR in both the 65 dBA and 85 dBA noise conditions. For each condition, participants could listen to the processed sentence as many times as they wished, and in any order, before providing their ratings. Responses were collected on a five-point Likert scale (1 = strongly disagree, 5 = strongly agree). For listening effort, 1 indicated very high effort, and 5, very low effort; for clarity and naturalness, 1 indicated very poor quality, and 5, excellent quality.

### 2.5. Sound Exposure Assessment

Sound exposure was evaluated on an acoustic manikin after the HINT and subjective rating tasks. To ensure comparability across conditions, a single sentence (“J’aime les couchers de soleil”) was used. The sentence was mixed with noise set at either 65 or 85 dBA, and presented at three SNRs (-4, 0, and +4 dB). Each noise and sentence combination was processed with each one of four signal processing configurations: NoAmp, LinAmp, Wdrc, DnrWdrc. This resulted in 24 unique stimuli (2 levels × 3 SNRs × 4 signal processing algorithms). Each configuration was presented once, and the signal sent to the receiver was recorded for every participant.

Recordings were post-processed to obtain an estimate of noise exposure using the following procedure. First, the receiver input signal was converted to Sound Pressure Levels (SPL) at the eardrum position using a receiver-to-ear-simulator transfer function measured on a HATS GRAS 45BB7 (Holte, Denmark) with the same earpiece as used by the participant. Since occupational safety standards express noise exposure limits at the theoretical position of the worker’s head in an undisturbed field, rather than in the worker’s ear canal, an eardrum-to-diffuse field correction was applied according to the ISO 11904-1 standard (International Organization for Standardization - Acoustics, 2002) and following the recommendations of Bonnet (2019). Finally, noise exposure was quantified as the equivalent continuous A-weighted level computed over the duration of each individual sentence.

Exposure levels across all 24 test conditions were compared to analyze the effect of different signal processing techniques and hearing profiles on the estimated noise exposure. Analyses were performed for the participant’s most impaired ear, as this ear required greater amplification and was expected to yield the highest effective noise exposure. Interactions between SNR and processing algorithms, as well as interactions between noise presentation levels and algorithms, were examined to evaluate how hearing profile and signal processing strategy affect noise exposure.

### 2.6. Statistical Analysis

All statistical analyses were performed in R version 2024.04.2+764 (Vienna, Austria). First, descriptive statistics were calculated to provide information on the participants’ groups, including means and standard deviations for age and PTA_4_, as well as counts and proportions for sex.

Then, separate linear mixed-effect models were built for each dependent variable: *SRTs*, subjective *listening effort* and *voice clarity and naturalness* ratings, as well as *noise exposure* estimates. Independent variables included the type of signal processing, presentation levels and their interactions. For the noise exposure models, SNR was included as a fixed factor. Participants were included as a random intercept to account for within-subject repeated measures. Models were fitted using the restricted maximum likelihood (REML) criterion. Model residuals were inspected to verify the assumption of normality, and when deviations were observed, a Box–Cox transformation was applied (Box & Cox, 1964). Statistical significance was interpreted hierarchically. First, the models with interaction were tested. If an interaction was significant, post-hoc tests were conducted; otherwise, the main effects were examined separately. For significant main or interaction effects, post-hoc pairwise comparisons were carried out using Tukey’s Honestly Significant Difference (HSD) adjustment to control for multiple testing (Tukey, 1949). For all analyses, a significance level of 5% was adopted.

Results are presented separately for each main dependent variable: *SRTs*, subjective *listening effort* and *voice clarity and naturalness* ratings, and *noise exposure* estimates. Analyses were performed independently for participants with and without hearing loss (HL and NH groups, respectively).

## 3. Results

### 3.1. Group Characteristics

Participants were divided into two groups based on the PTA_4_ for their better ear, which was used for all analyses except for the noise exposure assessment. Noise exposure was assessed using the worse ear, i.e. the ear with the largest amplification needs.

**Figure 5.**
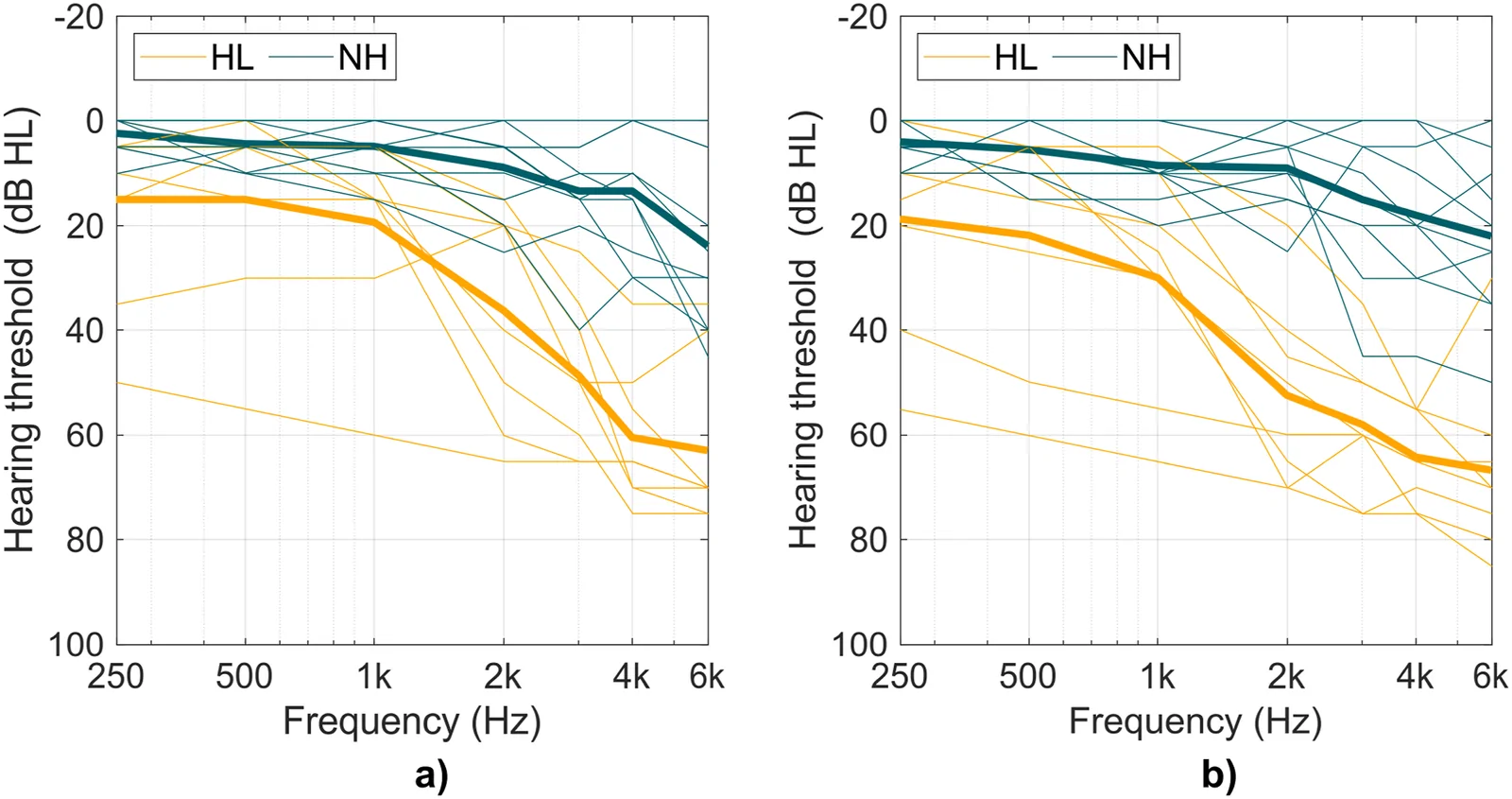
(Color online) Hearing thresholds from 250 Hz to 8 kHz for individual participants (thin lines) and group means (thick lines) based on the participants’ (A) better ear and (B) worse ear. Units are decibels hearing level (dB HL).

The HL group was composed of 8 participants, one female and 7 males. The NH group was composed of 10 participants, 3 males and 7 females. Figure 5 shows the individual audiograms and group mean audiograms for participants’ least impaired and most impaired ear. The mean and standard deviation for the participants’ PTA_4_ best ear and worst ear are referenced. Each group characteristics are summarized in Table 1.

### 3.2. Speech Reception Threshold

**Figure 6.**
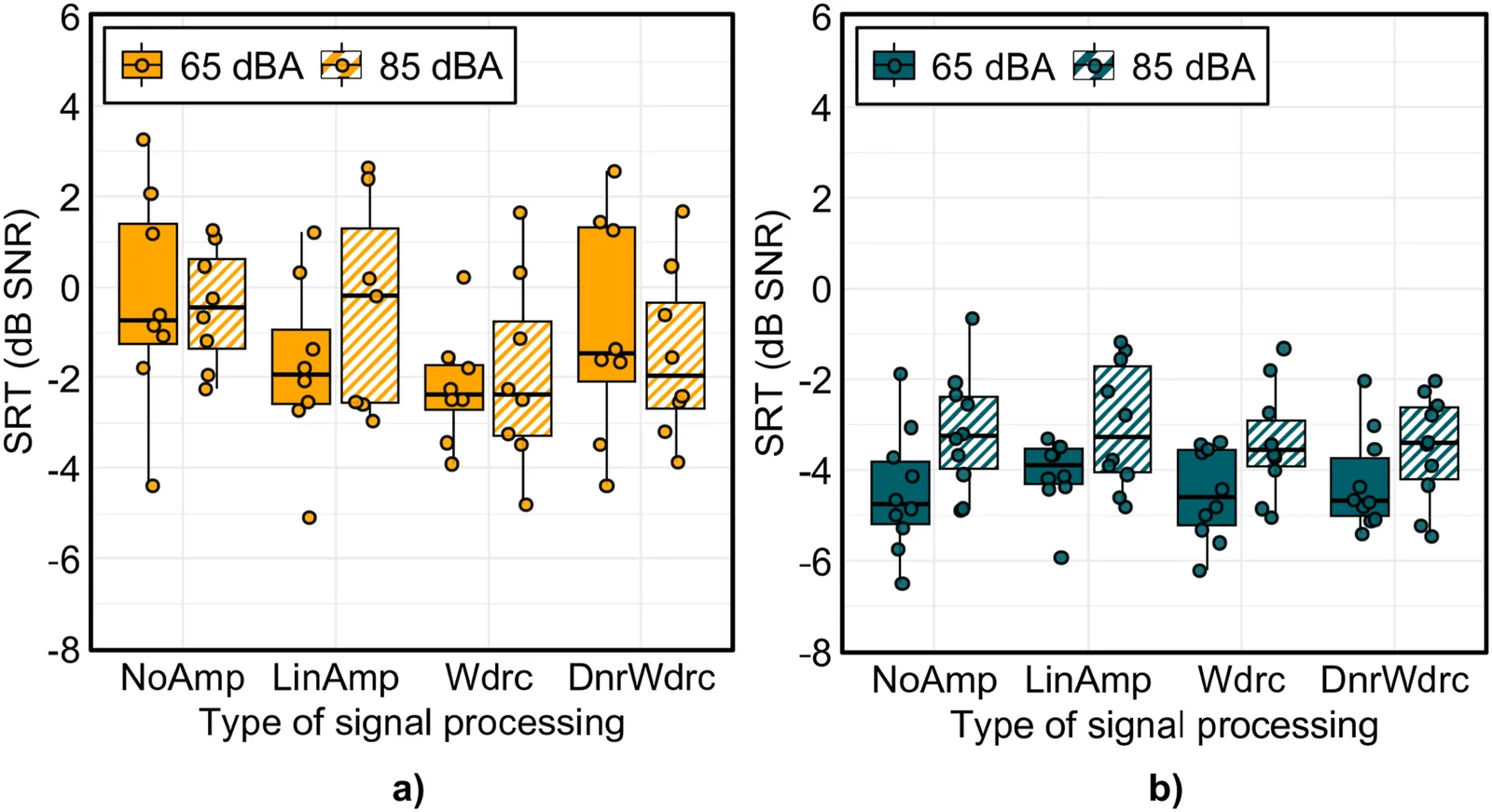
(Color online) *SRTs* for each type of signal processing and presentation level for (A) the HL group and (B) the NH group. Circles denote the individual results. Better performance is characterized by a lower *SRT.*

*SRTs* for the two groups, and for each noise presentation level and type of signal processing, are presented in Figure 6.

For the HL group, the interaction between presentation level and type of signal processing did not significantly affect *SRTs*. There was a main effect for the type of signal processing on *SRTs* (*p* < 0.001), but not for noise presentation level. Post-hoc analyses indicated that participants performed significantly better with WDRC than without any amplification, i.e., with compression limiting alone (*p* < 0.001). The other signal processing techniques led to small but not statistically significant improvements in *SRT* relative to compression limiting alone. Differences between WDRC and linear amplification were not statistically significant, although mean *SRTs* were consistently better for WDRC at both 65 dBA and 85 dBA. No significant improvement was found with combined WDRC and DNR compared to WDRC alone. These results indicate that, for listeners with hearing loss, WDRC provided the most consistent benefit for speech intelligibility in industrial noise and additional use of DNR did not yield further measurable gains or loss in intelligibility.

For the NH group, the interaction between level and type of signal processing also did not significantly affect *SRT* scores. There was no main effect of signal processing strategies. A significant main effect of the noise level was found (*p* < 0.001), with poorer performance at 85 dBA than at 65 dBA. This suggests that for normal-hearing participants, increasing background noise level degraded speech intelligibility regardless of amplification strategy.

### 3.3. Subjective Ratings of *Listening Effort* and *Voice Clarity and Naturalness*

**Figure 7.**
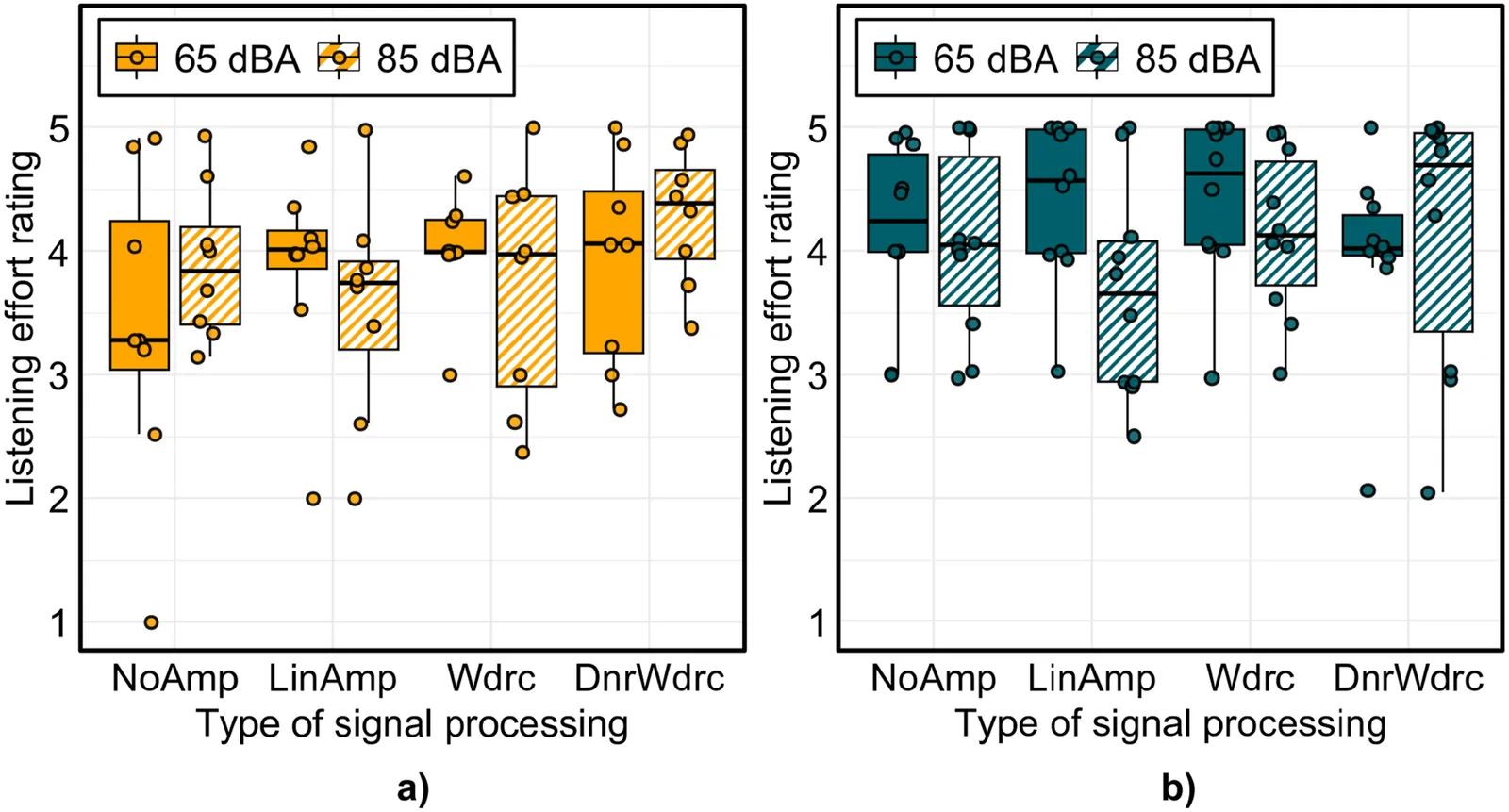
(Color online) Boxplots of *listening effort* ratings for (A) the HL group and (B) the NH group for each presentation level and signal processing strategy. Circles denote the individual results. Ratings were given on a 5-point Likert scale (1 = high effort, 5 = low effort) for presentation levels of 65 dBA and 85 dBA.

**Figure 8.**
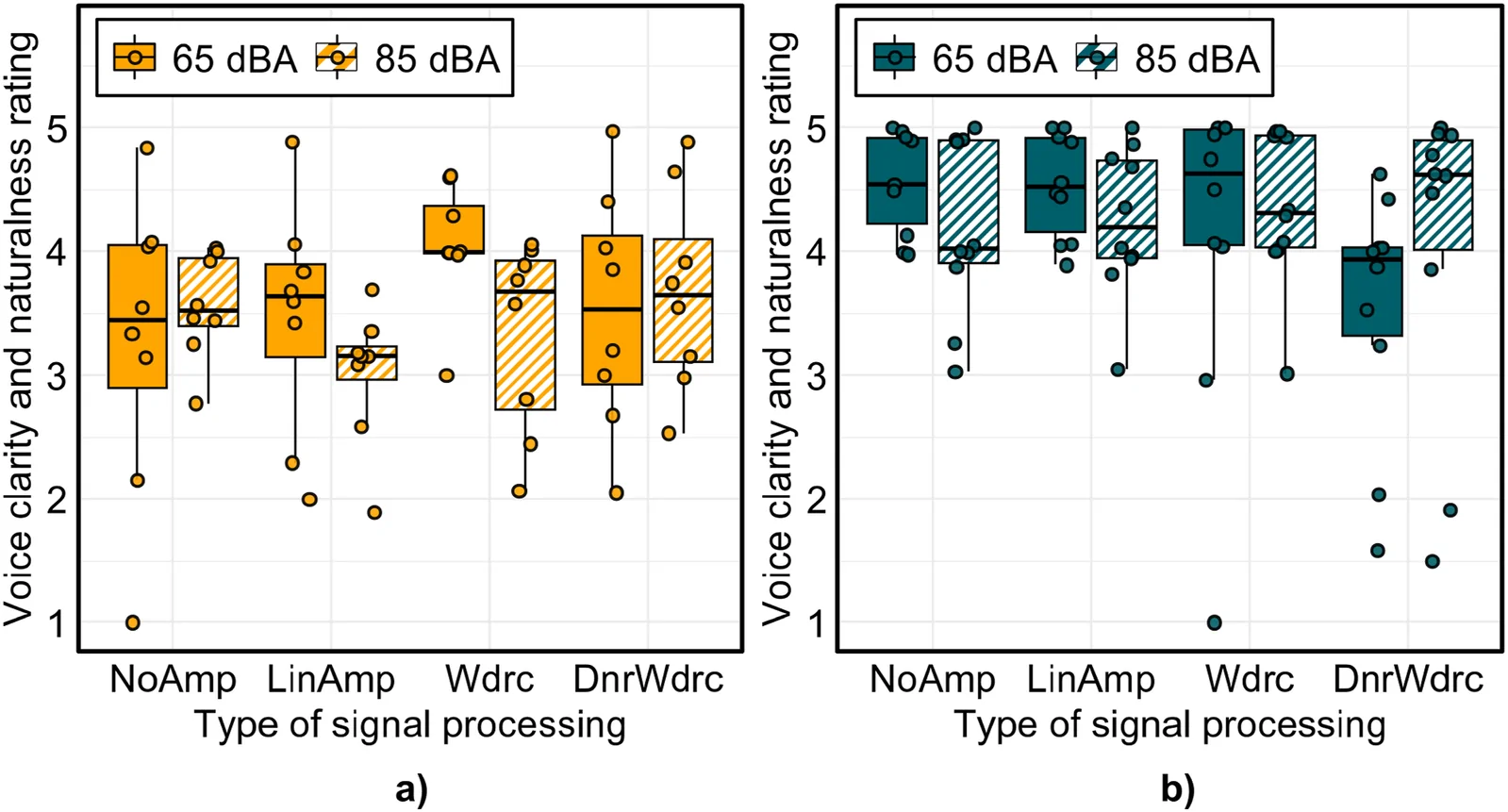
(Color online) Boxplots of *voice clarity and naturalness* ratings for (A) the HL group and (B) the NH group for each presentation level and signal processing strategy. Circles denote the individual results. Ratings were given on a 5-point Likert scale (1 = high effort, 5 = low effort).

The summary of ratings for each group is presented in Figure 7 for *listening effort* and in Figure 8 for *voice clarity and naturalness*.

For the HL group, the interaction between signal processing type and noise presentation level was not significant for *listening effort* ratings. Neither signal processing type nor presentation level showed a significant main effect. For *voice clarity and naturalness* ratings, the interaction between signal processing type and presentation level was not significant. Neither factor showed a significant main effect.

For the NH group, there was no significant interaction between presentation level and type of signal processing for *listening effort* ratings. Neither presentation level nor signal processing showed a significant main effect. Similarly, neither type of signal processing, nor presentation level, nor the interaction had a significant effect on *voice clarity and naturalness* ratings.

### 3.4. Noise Exposure

**Figure 9.**
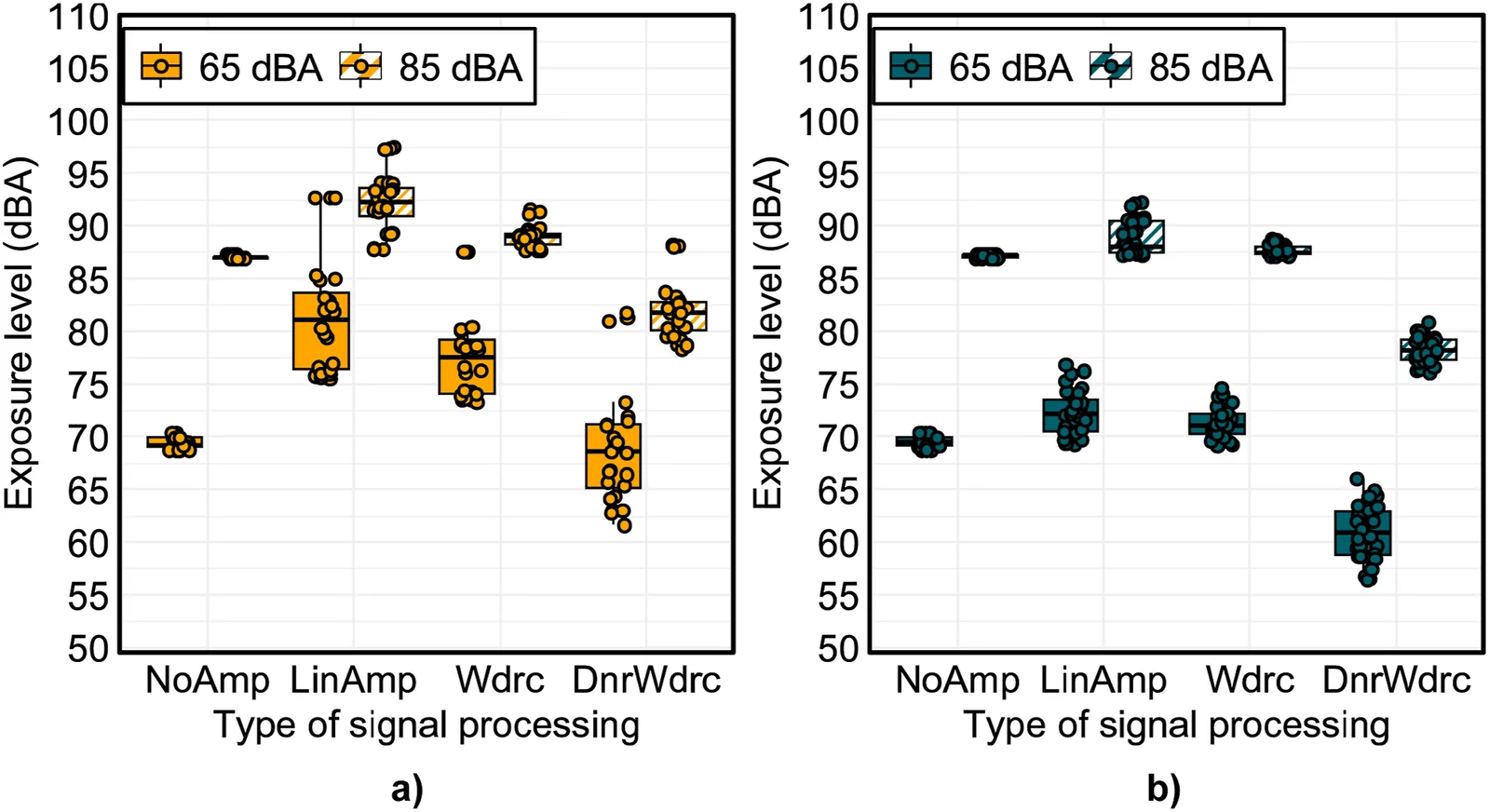
(Color online) Boxplot representation of the approximate exposure level (in dBA) across signal processing strategies and noise presentation levels, for all SNRs, for participants of (A) the HL group, (B) the NH group. Circles denote individual results by participant and by SNR.

**Figure 10.**
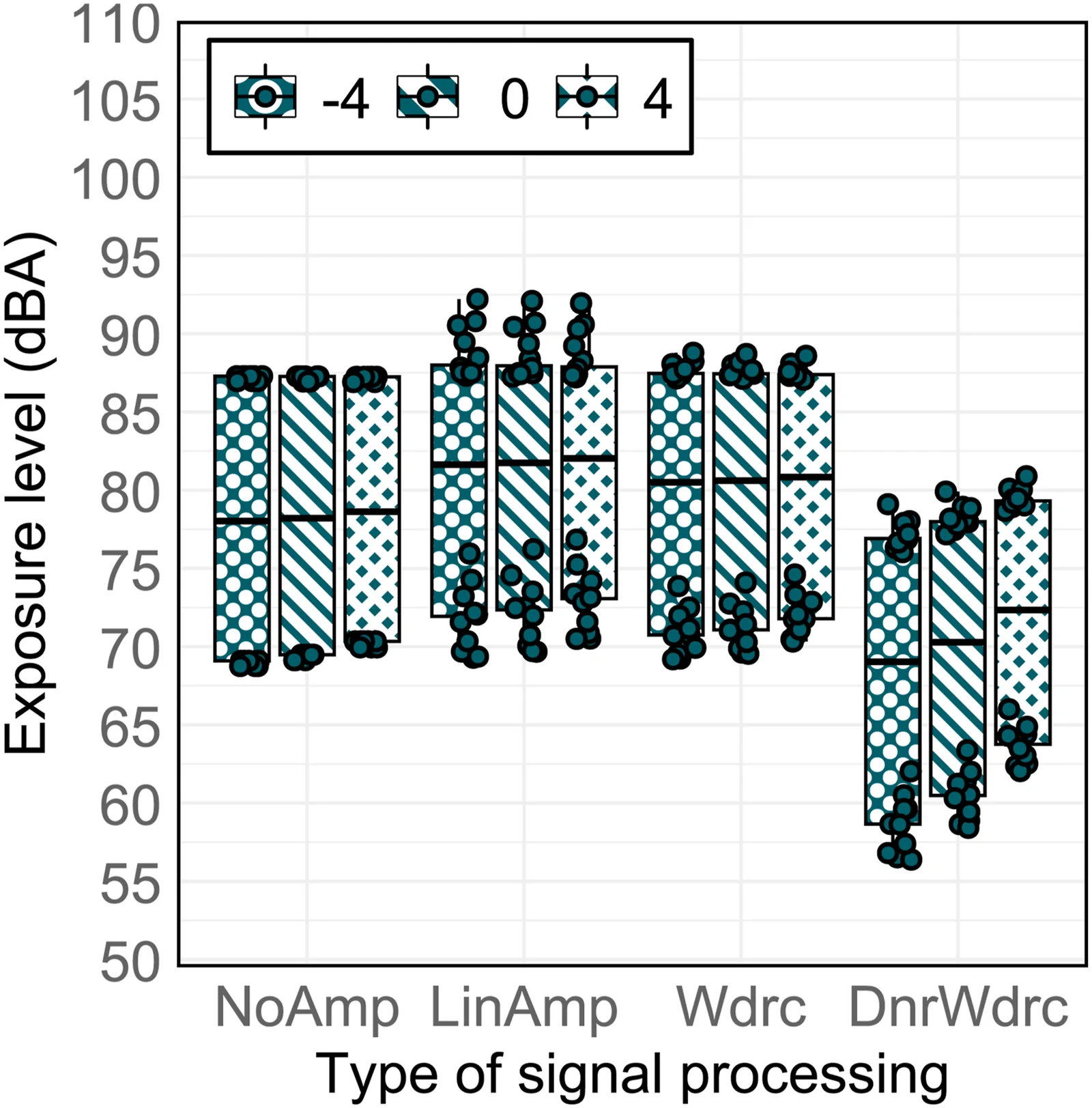
(Color online) Boxplot representation of the approximate exposure level for each signal processing strategy and SNR, for all noise presentation levels, for participants of the NH group. Circles denote individual results by participant and by noise presentation level.

Estimated A-weighted levels for each signal processing strategy, SNR, and noise level are shown in Figures 9 and 10. Because stimuli were reproduced through in-ear receivers rather than processed through a physical HPD, estimated exposure levels reflected the characteristics of the electronic sound pathway, expected to dominate over the passive acoustic pathways at the tested levels. For the HL group, there was no significant interaction between SNR and type of signal processing used for *noise exposure*. However, there was a significant interaction (*p* < 0.001) between signal processing type and noise presentation level (Figure 9A). Across all signal processing types, *noise exposure* levels were significantly higher at 85 dBA than at 65 dBA (*p* < 0.001 for all comparisons). At 65 dBA, WDRC significantly increased exposure compared to processing without amplification (*p* < 0.001), whereas at 85 dBA the difference was small and not significant. Relative to linear amplification, WDRC significantly reduced exposure at both levels (*p* < 0.001). Adding DNR to WDRC further decreased exposure compared to WDRC alone (*p* < 0.001). Linear amplification produced higher *noise exposure* levels than all other types of signal processing at both levels (all *p* < 0.001). The combination of WDRC and DNR significantly reduced exposure at 65 dBA compared to both WDRC alone and linear amplification (both *p* < 0.001), but did not differ from processing without amplification. At 85 dBA, the combination of WDRC and DNR yielded the lowest exposure, significantly below all other types of signal processing (*p* < 0.001 for each comparison).

For the NH group, interactions between SNR and signal processing strategy, and between noise presentation level and signal processing strategy, were significant (both with *p* < 0.001). These results are illustrated in Figure 9B) and in Figure 10. Across all signal processing techniques, noise exposure levels were significantly higher at 85 dBA than at 65 dBA (*p* < 0.001 for all comparisons). At 65 dBA, WDRC produced significantly greater exposure than no amplification (*p* < 0.001) but there was no significant difference at 85 dBA. Relative to linear amplification, WDRC significantly reduced exposure at both levels (*p* < 0.01 at 65 dBA; *p* < 0.001 at 85 dBA). Adding DNR to WDRC further reduced exposure compared to WDRC at both levels (*p* < 0.001). Linear amplification consistently led to the highest exposure, exceeding all other types at both 65 dBA (*p* < 0.001 compared to WDRC combined with DNR and to processing without amplification; *p* < 0.01 compared to WDRC) and 85 dBA (*p* < 0.001 for all comparisons). WDRC with DNR consistently resulted in the lowest exposure at both levels (*p* < 0.001 for each comparison). Only WDRC combined with DNR showed significant variation in exposure across SNRs. For combined WDRC and DNR, exposure decreased as SNR increased: from –4 dB to 0 dB and +4 dB (*p* < 0.0001 for each step). Within each SNR, all signal processing strategies differed significantly from each other, with *p*-values ranging from <0.05 to <0.001 . Linear amplification always yielded the highest exposure level, followed by WDRC, then no amplification, and finally the combination of DNR and WDRC.

## 4. Discussion

In the following section, the results are interpreted in relation to the study’s dual objectives: investigating the effects of typical hearing aid signal-processing algorithms on speech intelligibility for listeners with varying hearing abilities, and evaluating their influence on overall noise exposure.

### 4.1. Speech Intelligibility

#### 4.1.1. Effect of Noise Presentation Level

The higher SRTs at 85 dBA noise than at 65 dBA indicate a decrease in speech intelligibility for participants without hearing loss. This is consistent with previous findings reporting reduced speech recognition at high noise presentation levels, possibly due to the broadening of auditory filters and to the associated upward spread of masking (i.e., the masking of higher frequency sounds by lower frequency sounds) (Goshorn & Studebaker, 1994; Hornsby & Ricketts, 2001; Studebaker et al., 1999). Note that for NH participants, the compression limiter, which was set at 90 dBA, was hardly engaged at either presentation level and likely did not play a major role in the observed difference. The trend for decreased speech intelligibility at 85 dBA was not observed for participants with hearing loss. One explanation lies in the signal levels reaching the ear. When amplifying the signal to match the participant’s hearing loss, an input signal presented in 65 dBA ambient noise could result in levels above 80 dBA at the eardrum, as observed in the noise exposure results, while the use of compression limiting maintained signals presented in the 85 dBA ambient noise close to 90 dBA. As both scenarios involve relatively high presentation levels, similar performance across noise presentation was found.

However, this does not explain the similar performance in the condition without amplification nor DNR where participants with hearing loss performed similarly at 85 dBA and 65 dBA noise presentation levels. This does not necessarily imply that participants with hearing loss are unaffected by elevated presentation levels. On the contrary, once audibility is accounted for, their speech intelligibility appears similarly affected by increasing presentation level (Hornsby & Ricketts, 2001; Studebaker et al., 1999). Individuals with hearing loss may need higher presentation levels for speech to be audible and thus may not perform as well at lower presentation levels. These findings demonstrate that presentation level alone does not fully explain speech intelligibility performance of hearing-impaired listeners, as effective audibility needs to be accounted for.

#### 4.1.2. Effect of Amplification Strategy

For participants in the HL group, only the WDRC algorithm led to a significant improvement in SRTs compared to the processing without amplification, while linear amplification did not yield significant differences compared to processing without amplification. This result partly aligns with a study of Bentler and Duve (2000), who compared linear and compression-based hearing aids using the HINT for listeners with mild to moderate hearing loss. The authors reported no significant differences between unaided and aided conditions for either amplification type. They attributed this finding to the noise presentation level used for the HINT (65 dB SPL), which may have provided sufficient audibility in the unaided condition, thereby limiting the potential benefit of amplification. However, in the same study, when using the Speech in Noise (SIN) test (Fikret-Pasa, 1993) at multiple presentation levels, they found that hearing aids significantly improved speech intelligibility at lower levels (53 and 63 dB SPL). At higher levels (83 and 93 dB SPL), performance was similar across all hearing aids and the unaided condition, with the linear hearing aid even producing poorer results at the highest level.

There was no significant differences in speech intelligibility between WDRC and linear amplification in the present study. Previous studies reported similar findings, showing comparable speech intelligibility outcomes for the two algorithms, especially at mid presentation levels (Bentler & Duve, 2000; Boike & Souza, 2000; Larson et al., 2000; Souza, 2002), which is expected as the gain at 65 dBA was very similar when using linear amplification or WDRC. Interestingly, at higher presentation levels, some studies have reported a slight advantage of WDRC over linear amplification (Bentler & Duve, 2000; Larson et al., 2000; Souza, 2002). Even though both amplification conditions may include compression limiting, because WDRC provides less gain at high presentation levels, the compression limiting algorithm is likely engaged less frequently than with linear amplification, therefore potentially introducing less distortion. While such a trend was observed for the 85 dBA noise presentation level, it did not reach significance, likely reflecting the limited sample size (*n*=8), inter-individual variability, or specific methodological factors, including the use of industrial noise, the chosen compression settings, and the HINT as the test. It should be noted that the amplification types used identical compression limiting parameters and that both linear amplification and WDRC applied frequency-dependent gains depending on the participant’s hearing loss. Thus, the use of a recorded non-stationary industrial noise may have influenced the activation of level-dependent amplification differently than for the typical speech-shaped noise used in other studies. Despite these limitations, this study extends previous work by evaluating algorithmic performances under occupational constraints in an industrial noise condition. Because only one type of industrial noise was studied, the results cannot be generalized to all industrial noise. Future studies should include different types of industrial noise.

No significant differences across signal processing techniques were found for the NH group. The prescription algorithm applied gain whenever thresholds exceeded 20 dB HL at any frequency, regardless of the overall pure-tone average. Consequently, some NH participants received minor amplification at some frequencies, which may have offered small benefits while introducing distortion through WDRC or compression limiting. The results for the NH group are consistent with a previous study where WDRC did not affect SRTs in stationary noise for normal-hearing individuals (Rhebergen et al., 2017).

Overall, our results suggest that WDRC can provide benefits for speech intelligibility in challenging industrial noise environments, though its advantage over linear amplification remains modest, especially at mid presentation levels. The benefits of WDRC are influenced by numerous factors, such as the choice of compression parameters, the number of channels, the type of noise, participant’s characteristics, and the use of a noise reduction algorithm (Ollivier et al., 2025; Souza, 2002). For example, earlier studies have reported clear benefits of WDRC in noise compared to linear amplification. Those studies used two-channel compression systems and the authors have reported that this limited number of channels might provide a benefit compared to a higher number of channels, responsible of spectral flattening (Laurence et al., 1983; Moore et al., 1992).

#### 4.1.3. Effect of Digital Noise Reduction

For both groups, no significant improvements in SRT were found when using the DNR algorithm. This result aligns with previous studies showing that traditional DNR implementations do not yield measurable benefits in speech intelligibility (Brons et al., 2013, 2014; Desjardins & Doherty, 2014; Hu & Loizou, 2007; Kates, 2017). These studies suggest that noise reduction often introduces distortion and artifacts that alter typical speech cues, especially at low SNRs, therefore limiting intelligibility improvements.

While benefits of noise reduction algorithms for speech intelligibility are limited, many studies have reported an improvement in perceived listening effort and noise annoyance (Brons et al., 2013, 2014; Desjardins & Doherty, 2014; Sarampalis et al., 2009). In the present study, although not statistically significant, the combination of WDRC and DNR was rated slightly better than the condition without amplification or DNR at both noise presentation levels for the HL group, and at 85 dBA for the NH group. The HL group also rated the combination of WDRC and DNR slightly better than WDRC alone at a higher noise level. These results suggest a small advantage in perceived listening effort when combining WDRC with DNR. The difference between our results and the clearer benefits of DNR reported in the literature may be due to methodological factors, such as question wording, signal processing characteristics and choice of compression parameters (Ollivier et al., 2025; Souza, 2002).

#### 4.1.4. Other Considerations in Assessing Speech Intelligibility in Noise

The speech stimuli used in our study were the original speech stimuli used for the HINT (Vaillancourt et al., 2005). These were recorded in quiet and, as a result, do not include the characteristics of ”Lombard speech”. The vocal effort when communicating increases as a function of ambient noise level and distance (Bouserhal et al., 2017) and speech produced with high effort has different characteristics than normal speech (Bouserhal et al., 2016). Further research should include speech stimuli that take into account this Lombard effect to match more realistic listening conditions. For example, stimuli in the *Spear* database consist of HINT sentences recorded in adverse noise conditions (Bouserhal et al., 2019).

In occupational settings, while speech intelligibility is important for effective communication, access to other critical signals, such as alarm or machinery sounds, is also essential for workers’ safety. DNR, which aims to reduce non-speech energy, may attenuate important environmental cues. In contrast, active noise reduction (ANR), which primarily targets low-frequency noise below 1000 Hz, may reduce upward spread of masking and facilitate the detection of warning signals (Casali et al., 2004). However, this benefit depends on the spectral characteristics of both the noise and the signal, as well as the attenuation profile of the hearing protection device. While Casali et al. (2004) reported improved comfort and enhanced alarm detection, their study involved normal-hearing participants, and the potential benefits for individuals with hearing loss remain uncertain. Future studies should evaluate different passive and active noise reduction strategies using additional outcome measures, such as alarm detection and sound localization, alongside speech intelligibility and noise exposure. In parallel, combining noise reduction and automatic warning signal detection (Bernstein et al., 2014; Carbonneau et al., 2013) offers a promising avenue to reduce noise while preserving access to critical auditory cues.

In the present study, speech intelligibility testing was conducted using only the hearing aid receivers, without incorporating the passive acoustic path through the earplug material itself (or the leakage around). In real-world conditions, sound reaches the eardrum both through the processed electroacoustic signal and through a passive sound pathway. Sound from this unprocessed passive pathway could interact with the amplified signal, particularly when the receiver output levels are reduced due to noise reduction for example. In addition, processing delays could lead to perceptible differences between the signals arriving via the receiver and the passive pathway (Lezzoum et al., 2016), impacting preference and potentially impacting speech intelligibility. The absence of this interaction in the current setup may therefore affect the generalizability of the results to real-world listening conditions. Future studies should aim to include both transmission pathways to better reflect realistic listening environments.

Additionally, challenges in recruiting participants, more specifically workers with hearing loss, resulted in a limited sample size. Even though the study had sufficient statistical power to detect the observed effects, it may not have been adequately powered to detect smaller or secondary differences that could also be relevant. Therefore, the findings should be interpreted with caution, particularly with regard to their generalizability. These findings should be confirmed with a larger and more diverse sample. Moreover, although no clear pattern was observed, future studies should formally examine the relationship between subjective ratings and speech intelligibility to better understand potential associations between perceived and measured performance.

### 4.2. Effect of Amplification Strategies on Noise Exposure

The noise exposure produced with different signal processing techniques gives a preliminary indication of potential overexposure caused by hearing aid amplification, in particular for high level industrial noise.

Among participants with hearing loss, WDRC limited increases in noise exposure under high-noise conditions while providing amplification at lower presentation levels. Indeed, WDRC increased the exposure level for the 65 dBA noise, compared to no amplification, as expected due to the prescribed gain at moderate levels. However, exposure levels in the WDRC and no-amplification conditions were comparable at 85 dBA, likely due to the lower WDRC gain at higher presentation levels (Figure 3). Linear amplification for the 85 dBA level consistently resulted in higher exposure levels than WDRC, as expected since gains are higher at high levels with linear amplification.

In most workplaces, 85 dBA is typically considered the level above which wearing hearing protectors is mandatory. The permissible 8-hour equivalent noise exposure level typically ranges between 80 dBA and 85 dBA, depending on national regulations and organizations guidelines. It is therefore crucial to accurately evaluate the noise generated inside the ear to assess the risk of overexposure when wearing devices with amplification capabilities. In this study, the exposure level produced by the research platform at a 65 dBA noise presentation level exceeded 85 dBA for three participants using linear amplification, whereas with WDRC only one participant was exposed to levels above 85 dBA. Participants with greater degrees of hearing loss are thus at risk of overexposure even in mid-level noise environments. This likely reflects the higher prescribed gain for individuals with more severe hearing loss, which, while beneficial for audibility, can substantially increase noise exposure. For the 85 dBA noise level, exposure levels with WDRC exceeded 85 dBA and slightly exceeded exposure levels observed with no amplification. These findings are consistent with previous research showing that hearing aids may lead to amplification beyond 90 dBA, and thus may put the user at risk of occupational overexposure (Dolan & Maurer, 1996).

Interestingly, combining WDRC with DNR resulted in lower exposure levels at high noise levels compared to WDRC alone. While WDRC alone provided a significant improvement in speech intelligibility, the associated exposure levels could be considered high, raising concerns about potential overexposure. From a workers’ safety perspective, combining WDRC with DNR appears to be a promising strategy to reduce noise exposure without compromising speech intelligibility.

Results on the noise exposure produced by the different signal processing types revealed distinct patterns for the NH group. WDRC increased noise exposure at mid-level presentation compared to no amplification, yet produced exposure levels similar to no amplification at higher presentation levels. The effect of the DNR algorithm on noise exposure depended on the presentation SNR. At lower SNRs, activating DNR led to reduced exposure levels, a trend that was statistically significant for the combined DNR and WDRC condition, while only a slight tendency was observed for other processing strategies. As SNR increased, signal energy also rose, which may partly explain this pattern. However, the fact that the trend was significant only with DNR suggests that the algorithm itself contributed to reducing noise exposure at low SNRs, consistent with its underlying principle: the more noise that is present in the signal, the greater the reduction applied. Nevertheless, disentangling the respective contributions of increased signal energy and DNR processing to the observed results remains challenging.

When the signal was processed with WDRC or linear amplification, participants in the NH group were similarly exposed than when the signal was processed without amplification. As those processing strategies did not lead to significant improvements in speech intelligibility, they may not be relevant for use by individuals without hearing loss. However, combining WDRC and DNR resulted in a significant decrease in exposure at both mid and high noise presentation levels. Thus, individuals without hearing loss could benefit from such algorithms for further protection without alteration of speech intelligibility.

In this study, DNR appears to function as a protection feature for workers with and without hearing loss. While promising, this result was obtained using a single noise condition, a wood-working factory noise. Previous studies have shown that DNR performance is influenced by the type of noise (Krishnamurthy & Hansen, 2013; Vihari et al., 2016), with better performance observed in stationary noise than non-stationary noises. Consequently, further research is needed concerning the extent to which the DNR algorithm used in this study may reduce noise exposure across a wide range of industrial noises.

Noise exposure in this study was limited to noise exposure produced by short speech sentences in background noise. The daily exposure of a worker wearing a device similar to the one presented in this study would be accumulated over an 8-hour period with various noise scenarios: speech in high noise, speech in low noise, high and low background noise only, etc. A correct estimation of the daily exposure should take into account all various exposure contexts. Also, the actual fit of the protection device, as well as the passive sound pathway, needs to be accounted for: additional tests in a full sound field are needed. Moreover, the attenuation provided by HPDs on real ears is different from the attenuation measured in a laboratory setting (Voix & Laville, 2009). In-ear dosimetry would make it possible to access more accurate noise exposure assessment (Bonnet, 2019).

## 5. Conclusions

This study examined the effect of various combinations of algorithms on speech intelligibility for listeners with and without hearing loss, and how these processing strategies influence overall noise exposure levels.

The results for the NH group showed no significant effect of hearing aid signal processing strategy on speech intelligibility. However, DNR may be a promising measure to reduce noise exposure while maintaining speech intelligibility at high noise levels.

WDRC can improve speech intelligibility in noise for individuals with hearing loss at the expense of greater noise exposure, unless the WDRC is combined with DNR. It appears to be risky to use linear amplification both in high level noise and in mid-level noise especially for individuals with higher degrees of hearing loss (mild to moderate in this study). WDRC may also lead to overexposure at moderate presentation levels for higher degrees of hearing loss (moderate to severe). Combining DNR and WDRC appears to be the best compromise for hearing aid use in noise for noise-exposed workers with mild to moderate hearing loss; noise exposure is significantly reduced and speech intelligibility not significantly altered.

The algorithms included in the study were designed to evaluate whether WDRC and linear amplification could benefit hearing-impaired workers in industrial noise. More advanced algorithms or self-adjusted prescription strategies might yield greater improvements in speech intelligibility while minimizing noise exposure. Because compression limiting can affect noise exposure and intelligibility by inducing distortion, the choice of the compression limiting threshold could be further studied to find a good compromise between safety and intelligibility. Future studies should also investigate how the interactions between WDRC, DNR, and compression limiting influence speech intelligibility and noise exposure at higher occupational noise levels, where SNR may be poorer, and across different types of industrial noise.

Future work should explore the performance of the device in full sound field mode, in high noise levels where both the electronic and passive sound pathway will contribute to the in-ear sounds and potentially impact speech intelligibility and noise exposure. Given this need, the integration of in-ear dosimetry with hearing aid algorithms seems promising to ensure that the measured noise exposure accounts for the passive sound pathway and for the varying device fits, amplification parameters and ear canal geometries. This real-time monitoring of the noise dose would allow the adjustment of amplification parameters or the compression limiting threshold during the workday, depending on the accumulated noise dose, without requiring workers to stop amplification or leave their environment. Such adaptive procedure could benefit both workers with and without hearing loss. Workers’ degree of hearing loss may also be accounted for in adjusting parameters to ensure adequate protection across individuals (Macrae, 1994). Such approaches would build on the present findings and help optimize both speech intelligibility and auditory safety in occupational settings. Finally, our findings support the need for further research on the use of hearing aids in occupational environments with high noise levels and on the need to exercise caution with use of hearing aids in noisy workplaces. Further work should also evaluate the performance of the integrated protective hearing aid device in this study against the use of level-dependent HPDs or the combination of a hearing aid and a passive HPD, as suggested by OSHA (Fairfax, 2004), in terms of both protection and situational awareness.
